# Association of Dementia Human Leukocyte Antigen (HLA) Profile with Human Herpes Viruses 3 and 7: An in silico Investigation

**DOI:** 10.29245/2578-3009/2021/3.1218

**Published:** 2021

**Authors:** Lisa M. James, Spyros A. Charonis, Apostolos P. Georgopoulos

**Affiliations:** 1 The HLA Research Group, Brain Sciences Center, Department of Veterans Affairs Health Care System, Minneapolis, MN, 55417, USA; 2 Department of Neuroscience, University of Minnesota Medical School, Minneapolis, MN 55455, USA; 3 Department of Psychiatry, University of Minnesota Medical School, Minneapolis, MN 55455, USA; 4 Department of Neurology, University of Minnesota Medical School, Minneapolis, MN 55455, USA

## Abstract

Human leukocyte antigen (HLA), the most highly polymorphic region of the human genome, is increasingly recognized as an important genetic contributor to dementia risk and resilience. HLA is involved in protection against foreign antigens including human herpes viruses (HHV), which have been widely implicated in dementia. Here we used an *in silico* approach^1^ to determine binding affinities of glycoproteins from 9 human herpes virus (HHV) strains to 113 HLA alleles, and to examine the association of a previously identified HLA-dementia risk profile^2^ to those affinities. We found a highly significant correlation between high binding affinities of HLA alleles to HHV 3 and 7 and the dementia risk scores of those alleles, such that the higher the estimated binding affinity, the lower the dementia risk score. These findings suggest that protection conferred by HLA alleles may be related to their ability to bind and eliminate HHV3 and HHV7 and point to the possibility that protection against these viruses may reduce dementia incidence.

## Introduction

The human leukocyte antigen (HLA) region is increasingly recognized as an important genetic contributor to dementia risk and resilience^2–10^. HLA plays a central role in host protection against foreign antigens such as those derived from pathogens including viruses and bacteria. HLA protection against such pathogens is facilitated by two main classes of HLA genes that code for cell-surface proteins that are instrumental in immune system responses to foreign antigens. Class I HLA molecules (HLA-A, B, and C genes) present intracellular antigen peptides to CD8+ cytotoxic T cells to signal destruction of infected cells whereas Class II HLA molecules (HLA-DR, DQ, and DP genes) present endocytosed extracellular antigen peptides to CD4+ T cells to promote B-cell mediated antibody production and immune memory. These two classes of HLA work in concert to facilitate pathogen elimination.

The effectiveness of HLA in pathogen elimination, however, hinges in part on the binding affinity between HLA proteins with those derived from a given pathogen. The HLA region is the most highly polymorphic in the human genome and subtle variations in HLA proteins have been shown to affect binding affinity thereby influencing disease outcomes^11,12^. Indeed, HLA has been implicated in more diseases than any other region of the human genome^13^ and is particularly implicated in autoimmune conditions^14^, and increasingly, in dementia^2–10^. With regard to dementia, we have used a population immunogenetic epidemiological approach in 14 Continental Western European countries to identify an HLA-dementia risk profile characterized by 127 HLA alleles that are either negatively correlated with the population prevalence of dementia and are therefore considered protective or HLA alleles that are positively correlated with the population prevalence of dementia and are presumed to promote susceptibility^2^. We hypothesized that HLA protection against dementia is related to superior binding affinity to harmful antigens and that, conversely, HLA susceptibility to dementia is related to persistent foreign antigens (due to poor binding affinity) that may directly damage cells and/or stimulate chronic inflammatory responses and autoimmunity^2^.

Numerous pathogens have been implicated in dementia^15,16^, perhaps none more so than human herpes viruses (HHV)^17–22^. Although HHV are nearly ubiquitous^23–25^, dementia is relatively rare; we have previously suggested that HLA variations may moderate the association of HHV infection and dementia such that some alleles may provide superior binding affinity to specific strains, thus exerting a protective role for dementia^1^. In an initial *in silico* investigation, we evaluated the binding affinity of three dementia-protective and three neutral Class II HLA alleles to HHV epitopes^1^. We found that the HLA alleles that were protective against dementia had significantly higher binding affinity to HHV epitopes than the neutral alleles, with the most significant differences found for HHV6 glycoproteins. Here we extend that line of research to evaluate the association between the dementia risk scores of a large number of HLA Class I and II alleles previously characterized in an HLA-dementia risk profile^2^ and the affinity of those alleles with glycoproteins from 9 HHV strains.

## Materials and Methods

### HHV proteins

The amino acid sequences of surface glycoproteins from HHV 1–8 (1, 2, 3, 4, 5, 6A, 6B, 7, 8) were retrieved from the UniprotKB database^26^. Table 1 gives the details of these proteins and associated information regarding the number of *n*-mers used in the analyses of Class I and Class II alleles.

**Figure 1.**
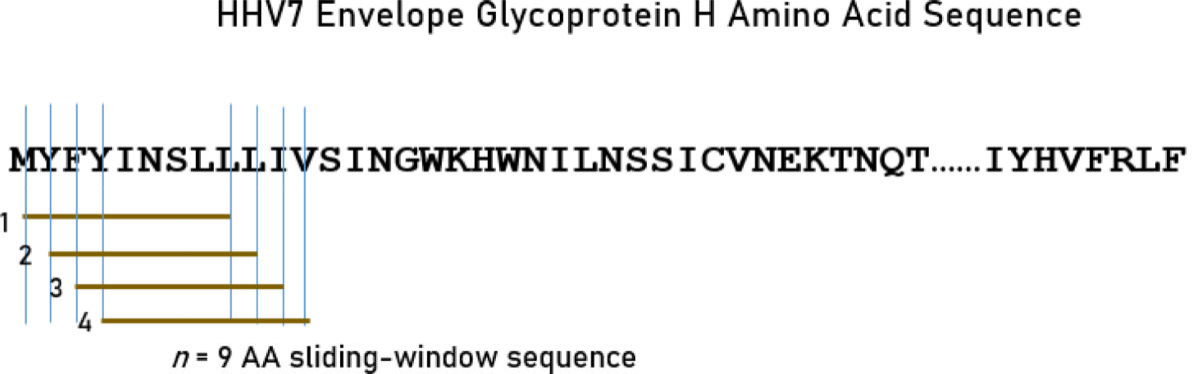
The sliding-window testing method is illustrated for 9-mers, as applied to the envelope glycoprotein H amino acid sequence of HHV7 to investigate binding affinities to HLA Class I molecules. Capital case letters in the sequence denote a specific amino acid residue. Brown horizontal lines indicate four 9-mer subsequences obtained by shifting the sliding window by one amino acid residue at a time.

**Figure 2.**
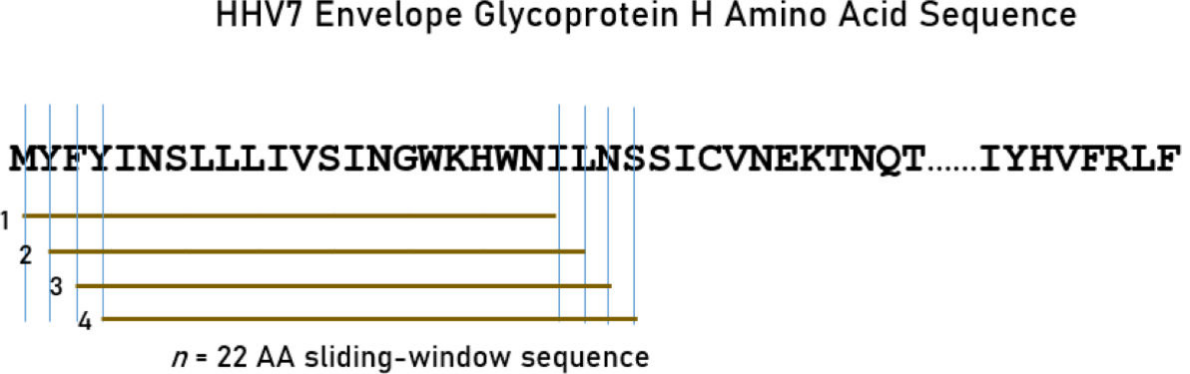
The sliding-window testing method is illustrated for 22-mers, as applied to the envelope glycoprotein H amino acid sequence of HHV7 to investigate binding affinities to HLA Class II molecules. Capital case letters in the sequence denote a specific amino acid residue. Brown horizontal lines indicate four 22-mer subsequences obtained by shifting the sliding window by one amino acid residue at a time.

A sliding window approach^1^ was used to partition the sequence of each glycoprotein into subsequences of 9-mers (for HLA Class I analyses; Fig. 1) and 22-mers (for HLA Class II analyses; Fig. 2) that covered the entire length of the protein. These *n*-mers were chosen given that they are typically short for Class I molecules (8–10 amino acids, AA) and around 20 for Class II molecules^27^. For each *n*-mer, a set of subsequences was generated (number of subsequences = length of glycoprotein – *n*). For both kinds of *n*-mers (*n*=9, Class I, and *n*=22, Class II), subsequences were collected and queried in the IEDB database (www.iedb.org) in order to identify potential epitope peptides that are recognized by and bind to HLA Class I and II surface receptor proteins. IEDB queries were performed for each of the sliding-window aggregated sequences against 113 HLA alleles (55 Class I and 58 Class II; see below). Binding affinity predictions were obtained using the NetMHCIIpan method^28^. For each *n*-mer, a binding affinity score was predicted and reported as a percentile rank by comparing the peptide’s score against the scores of five million random *n*-mers selected from the SwissProt database^26^; smaller percentile ranks indicate higher binding affinity (“good binders”). For each HHV strain and HLA allele, the average percentile rank of *n*-mer ranks <1 (APR<1) was used as a high binding affinity measure for quantitative analyses.

### HLA alleles

We used 113 HLA alleles out of 127 alleles for which we had obtained a dementia-HLA profile^2^ using data from 14 Continental Western European Countries (Austria, Belgium, Denmark, Finland, France, Germany, Greece, Italy, Netherlands, Portugal, Norway, Spain, Sweden, and Switzerland). Those 127 alleles comprised 69 Class I and 58 Class II alleles^2^; of those, in the present study we used all 58 Class II alleles (N = 15, 14, 29 for DPB1, DQB1 and DRB1 genes, respectively) but only 55 Class I alleles (N = 17, 25, 13 for A, B, and C genes, respectively) because 14 alleles could not be modeled by the NetMHCIIpan method.

### Dementia-HLA profile (dementia risk scores)

The dementia-HLA profile consisted of 113 Fisher z-transformed correlations ŕ between each HLA allele frequency and log-transformed dementia prevalence^2^. These values can be regarded as continuous-varying HLA-related dementia-risk scores, such that (a) their sign indicates protection (negative) or susceptibility (positive) to dementia, and (b) their absolute value indicates the strength of their effect (protection or susceptibility). Of the 113 alleles here, 51 were dementia-protective alleles (negative scores) and 62 dementia-susceptibility alleles (positive scores).

### Data analysis

The main objective of this study was to evaluate the association between specific HLA-HHV strain binding affinity and the HLA-dementia risk scores. For this purpose, we computed the Pearson correlation coefficient between the dementia risk scores and HHV-specific HLA affinity (APR<1) for each HHV strain. For the group of alleles with APR ≥ 1, i.e. alleles that were not used in the correlation analysis above, we tested the proportion of susceptibility alleles against the null hypothesis of the proportion = 0.5 using a one-sample binomial test of proportions. The IBM-SPSS statistical package (version 27) was used for these analyses.

## Results

**Figure 3.**
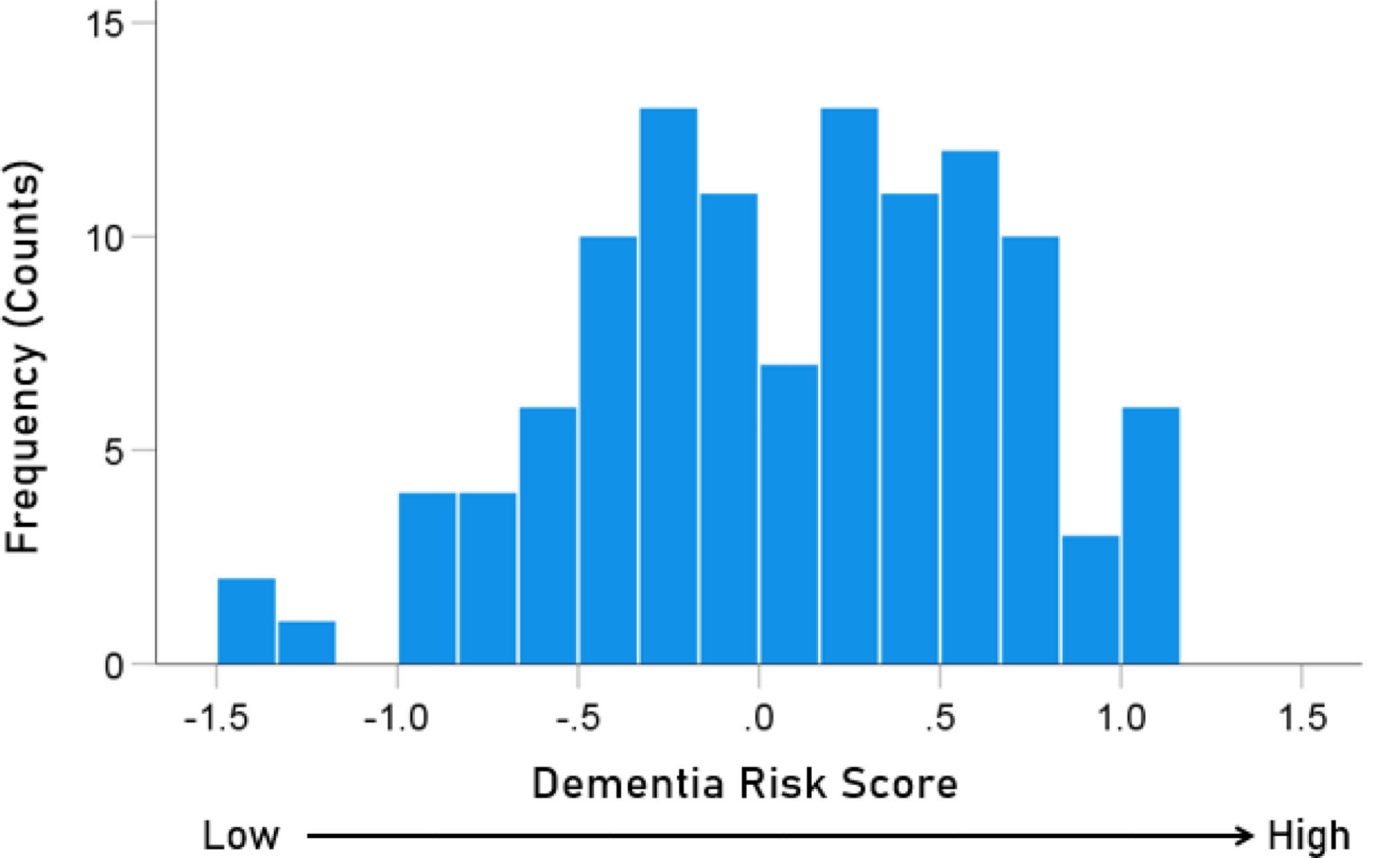
Frequency distribution of the dementia risk scores of the 113 alleles used (Table 2).

**Figure 4.**
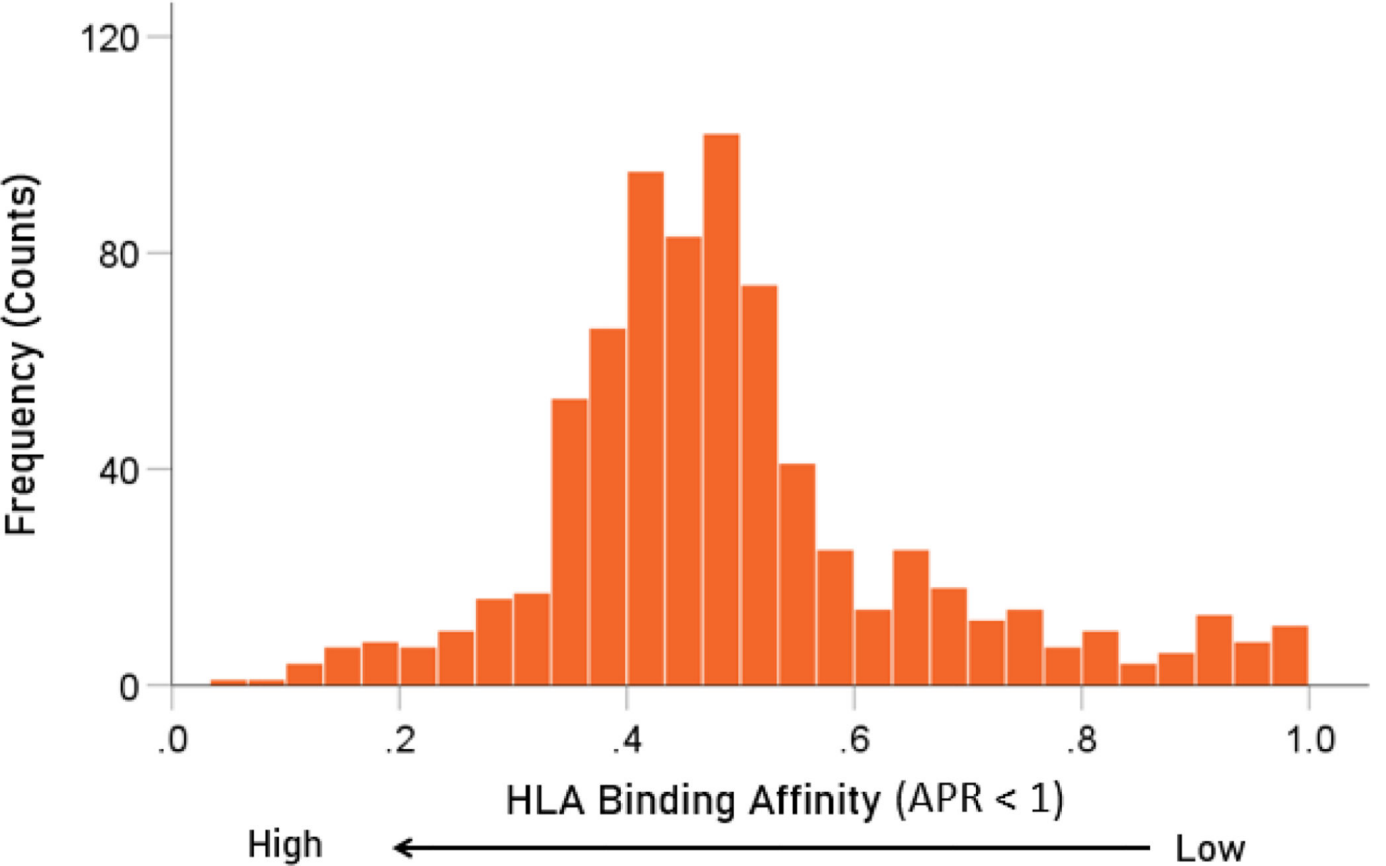
Frequency distribution of APR<1 values (N = 752 of a total of 9 HHV strains × 113 HLA alleles = 107 values; the remainder had values of APR≥1).

**Figure 5.**
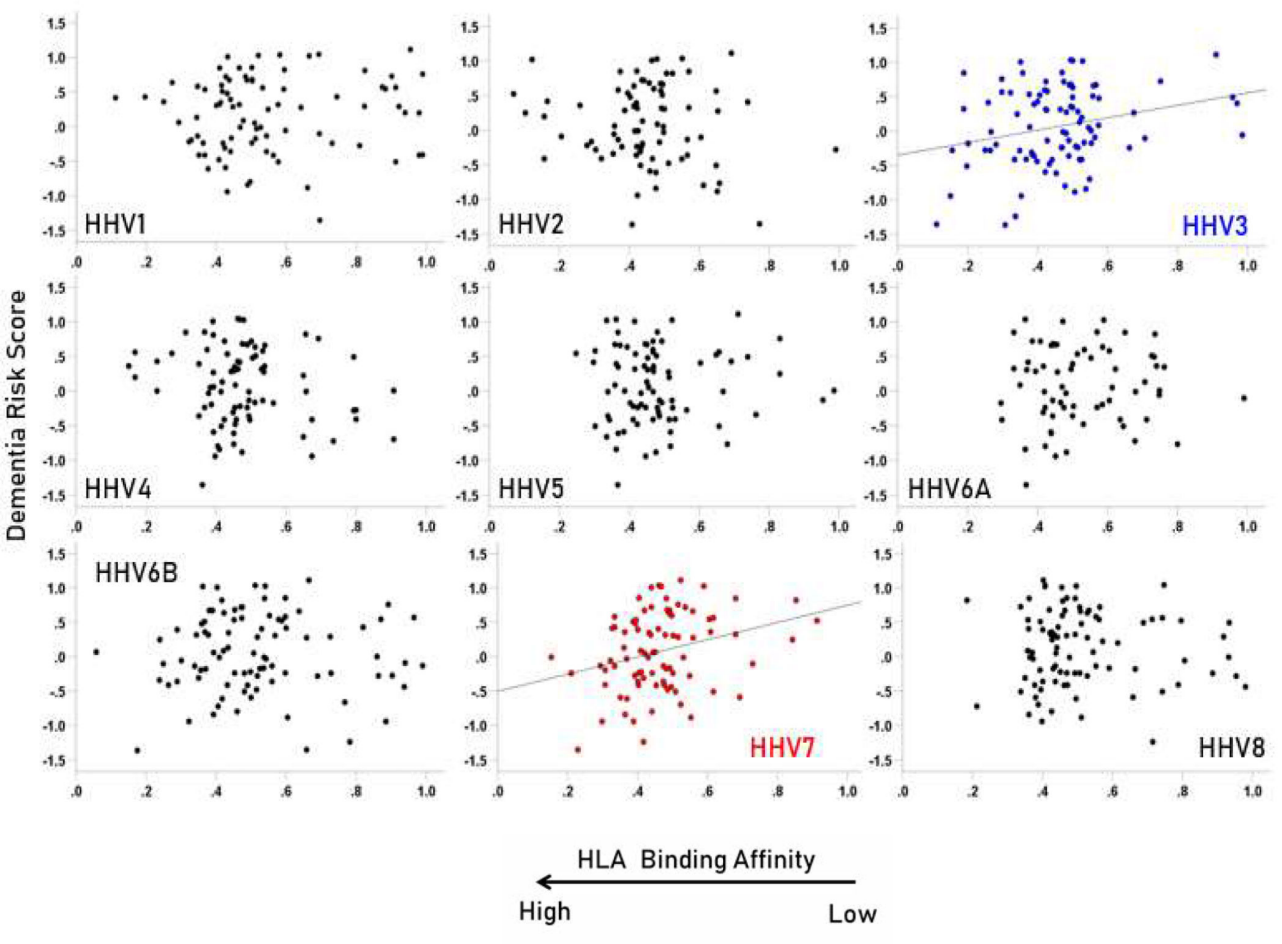
Scatter plots of dementia risk scores against APR<1 values for the each HHV strain. A statistically significant correlation was found only for HHV3 (r = 0.260, P = 0.013, N = 91) and HHV7 (r = 0.287, P = 0.008, N = 85). All correlations and associated statistics are given in Table 3.

**Figure 6.**
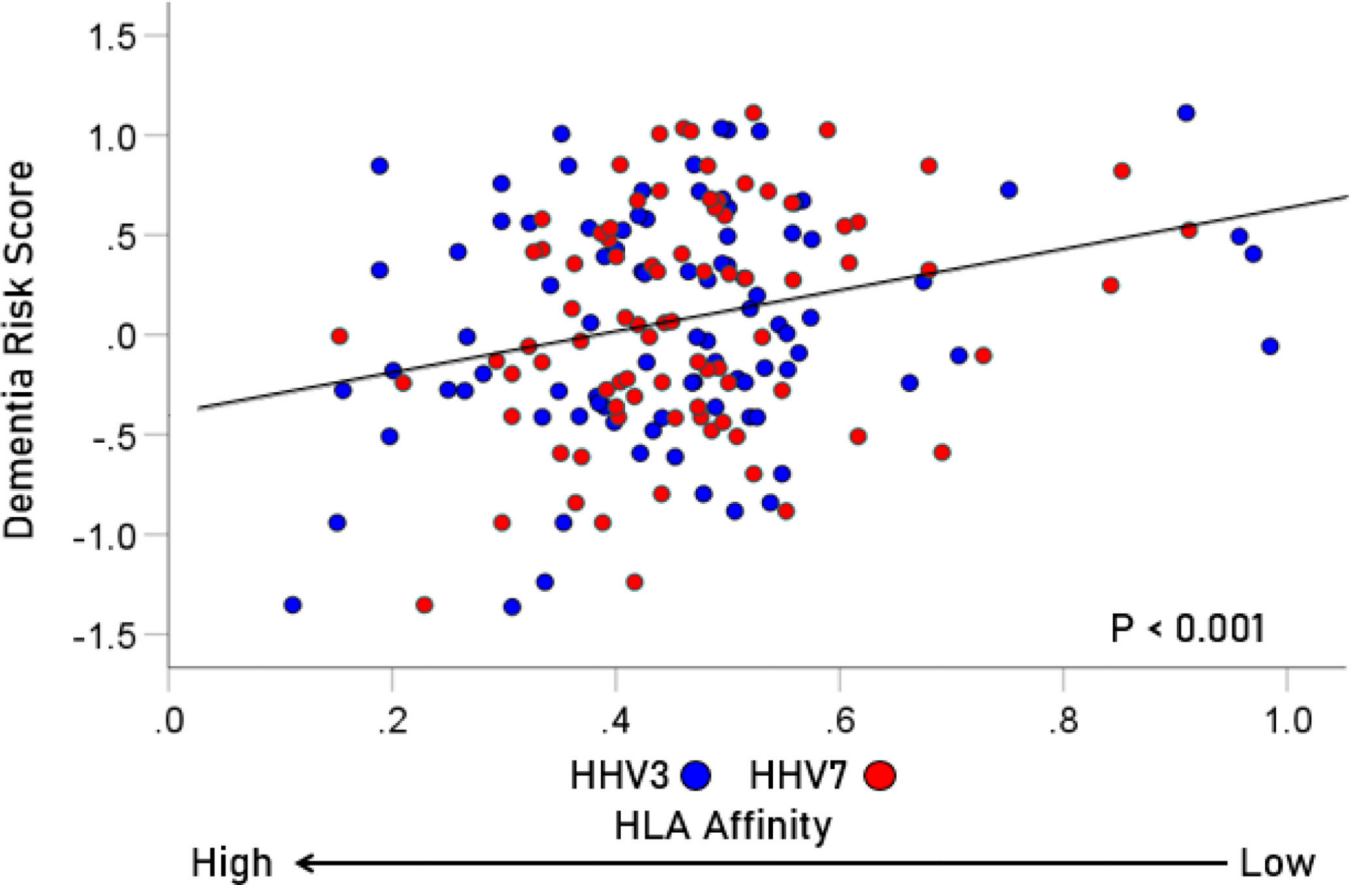
Scatter plot of dementia risk scores against APR<1 values for the pooled data of HHV3 and HHV7 (r = 0.270, P = 0.00029, N = 176).

The frequency distributions of the HLA-dementia risk scores and APR<1 values (all HHV strains) are shown in Figures 3 and 4, respectively. For each HHV strain, the dementia risk scores of individual alleles are plotted against the APR<1 value of the corresponding allele in Figure 5. The correlation coefficients and associated statistics for each HHV strain are given in Table 3. Statistically significant correlations were found only for HHV3 (blue in Fig. 5) and HHV7 (red in Fig. 5). The pooled APR<1 values for these two HHV strains are plotted in Figure 6; the Pearson correlation coefficient was 0.270 (P = 0.00029, N = 176).

For HHV3 and HHV7, there were a total of 2 × 113 = 226 alleles tested. Of those, 176 had values of APR < 1, and, hence were used for the correlation analysis above (Fig. 6). The remaining 50 alleles had values of APR ≥ 1 and were not eligible for the correlation analysis. However, we were interested to find out whether that group was enriched with susceptibility (positive) dementia risk scores, a hypothesis in line with the correlation findings above. Indeed, the proportion of susceptibility scores in that group was 33/50 = 66%, a proportion significantly higher than that of the null hypothesis of 50% (binomial one-sample proportions test, z = 2.263, two-sided P = 0.024).

## Discussion

In this *in silico* study we evaluated estimated binding affinities of glycroproteins from 9 HHV strains and 113 HLA alleles and examined the association of those affinities with a previously-identified HLA-dementia risk profile^2^. The findings indicated that for HHV3 and HHV7, but not for other HHV, the glycoprotein binding affinities were significantly associated with the dementia risk score such that higher binding affinity was associated with lower dementia risk. These findings suggest that protection conferred by dementia-protective HLA alleles is related, in part, to their ability to bind and eliminate HHV3 and HHV7. These findings add to the literature linking HHV to dementia and implicate HHV3 and HHV7, in particular, in dementia risk.

HHV3, often referred to as varicella-zoster virus (VZV), is a highly transmissible disease that results in chicken pox (varicella) upon initial infection, typically in childhood, and after a period of latency may reactivate as shingles (herpes zoster) in later adulthood. Acute varicella infection is usually mild, however, reactivation as herpes zoster is associated with several neurological complications including stroke, postherpetic neuralgia, meningitis, myelopathy, and ocular disorders^29–31^. Moreover, VZV infection has been associated with cognitive decline^32^ and dementia^33–36^. Among those infected, antiviral treatment has been associated with reduced dementia risk^33–36^, providing further evidence supporting a role of HHV3 in dementia pathogenesis. HHV3 is the only herpes virus for which effective vaccines exist – one to prevent varicella and another to prevent herpes zoster^31^. Introduction of vaccination programs has resulted in a rapid decrease in varicella^37^ and herpes zoster^38^ incidence. Furthermore, preliminary evidence indicates that shingles vaccination is associated with reduced incidence of dementia^39^.

HHV7 is an extremely common virus that affects most humans primarily during childhood with HHV7 seroprevalence reaching 76% in children aged 3–6^40^ and 98% in adults^41^. HHV7, which is commonly referred to as Roseolovirus along with genetically and biologically similar HHV6 variants^42,43^, is predominantly transmitted in saliva and is continuously shed in saliva of healthy adults^44^. Infection with HHV7 has been shown to result in cytopathic effects^43 44^, down regulation of CD4+ cells^45,46^, and altered trafficking of Class I MHC molecules^47^. Like other herpesviruses, HHV7 persists after the primary infection as a lytic or latent infection that can be reactivated, or can reactivate other HHV^48^, contributing to disease^42–44^. HHV7 has most widely been associated with exanthem subitum and pityriasis rosea^44^. In addition, HHV7 has been associated with neurological signs and symptoms^49^ and with demyelinating diseases^50^, and recently, with dementia^51^. Specifically, increased HHV7 was found in post-mortem brain tissue of patients with AD relative to controls, and HHV7 corresponded with hallmark AD-related neuropathological findings including amyloid beta deposition^51^. Notably, recent evidence suggests amyloid beta may be involved in innate immunity, exerting antimicrobial properties to HHV^52^.

The findings of the present *in silico* study provide novel and compelling evidence linking HHV3 and HHV7 to dementia. Considering the present findings in light of the discordant rates of HHV3 and HHV7 infection with dementia prevalence, we suggest that HHV3 and HHV7 may contribute to dementia and associated neuropathology in those lacking HLA alleles that are able to bind with sufficient affinity and immunogenicity to eliminate the virus. In the absence of immunogenetic protection against these viruses, prevention of HHV infection via vaccination may be of paramount importance for reducing dementia incidence. Indeed, previous studies have reported reduced dementia incidence following vaccination for influenza^53,54^ and tetanus, diphtheria, pertussis (Tdap)^55^. Preliminary evidence suggests a protective role of shingles vaccination against dementia^39^ though additional studies are needed to verify those initial intriguing findings. Approved vaccines do not yet exist for other HHV though several are under development, particularly for herpes simplex virus^56^. The present findings point to a potential benefit of HHV7 vaccine development in reducing dementia risk.

These novel findings, however, must also be considered in terms of several qualifications. First, the HLA-dementia profile at the center of these analyses were derived from Continental Western European countries and may not generalize to other regions given ethnic and geographic variation in HLA^57–59^. Future studies evaluating the associations of HLA-HHV affinity with HLA-dementia profiles in other populations will be important for corroborating and/or extending the present findings to other HHV strains and populations. Second, the present study only focused on HHV. Numerous microbes and viruses have been implicated in risk for dementia^15,16^. The same *in silico* approach used here can readily be applied to other infectious agents to evaluate their binding affinity with HLA alleles associated with dementia susceptibility and protection. Third, while this study identified HHV3 and HHV7 as playing a central role in HLA-mediated dementia, other non-HLA mechanisms that were not investigated here may influence the association of other HHV with dementia. Finally, we exclusively evaluated HLA-HHV glycoprotein binding affinity; binding affinity is one of many factors that contributes to disease outcomes. Additional in vitro and in vivo studies are warranted to further validate the findings of this *in silico* study and determine the relative influence of HLA-HHV binding affinity on dementia.

In summary, findings of the present study suggest that dementia risk scores, which are characterized by the sign and strength of the correlation between the population frequency of HLA alleles and the population prevalence of dementia, are significantly associated with HLA binding affinity to glycoproteins for HHV3 and HHV7. HLA alleles that bind with strong affinity to HHV3 and HHV7 presumably promote virus elimination, reducing deleterious downstream effects that may lead to dementia.

## Figures and Tables

**Table 1:** HHV proteins used. See text for details.

Virus	Protein description	UniprotKB ID	Protein length (AA)
HHV-1	Envelope glycoprotein D	Q69091	394
HHV-2	Envelope glycoprotein D	P03172	393
HHV-3	Envelope glycoprotein E	Q9J3M8	623
HHV-4	Envelope glycoprotein B	P03188	857
HHV-5	Envelope glycoprotein B	P06473	906
HHV-6A	Glycoprotein Q2	P0DOE0	214
HHV-6B	Glycoprotein Q1	Q9QJ11	516
HHV-7	Envelope glycoprotein H	P52353	690
HHV-8	Envelope glycoprotein H	F5HAK9	730

**Table 2:** Dementia-risk scores (r′)^2^ of the 113 HLA alleles used. See text for details.

	Allele	Dementia risk scores (r′)
1	A*01:01	−0.237
2	A*02:01	−0.593
3	A*02:05	0.853
4	A*03:01	−0.412
5	A*11:01	0.347
6	A*23:01	0.317
7	A*24:02	0.051
8	A*25:01	−0.031
9	A*26:01	0.478
10	A*29:02	0.131
13	A*31:01	0.357
14	A*32:01	0.580
15	A*33:01	−0.361
16	A*68:01	0.392
17	A*68:02	0.672
11	A*30:01	−0.220
12	A*30:02	−0.137
18	B*07:02	−0.941
19	B*08:01	−0.797
20	B*13:02	−0.240
21	B*14:02	0.719
22	B*15:01	−0.611
23	B*18:01	0.597
24	B*27:02	0.060
25	B*27:05	−0.195
26	B*35:01	0.306
27	B*35:03	0.672
28	B*37:01	−0.412
29	B*38:01	1.026
30	B*39:01	0.284
31	B*40:01	−0.509
32	B*40:02	−0.010
33	B*44:02	−0.841
34	B*44:03	0.086
35	B*49:01	0.847
36	B*50:01	0.509
37	B*51:01	0.535
38	B*52:01	0.317
39	B*55:01	−0.479
40	B*56:01	−0.309
41	B*57:01	−0.166
42	B*58:01	0.721
43	C*01:02	−0.011
44	C*03:03	−0.416
45	C*04:01	1.035
46	C*05:01	−0.174
47	C*06:02	−0.135
48	C*07:01	−0.362
49	C*07:02	−0.883
50	C*07:04	−0.238
51	C*12:02	0.636
52	C*12:03	1.007
53	C*14:02	0.681
54	C*15:02	0.660
55	C*16:01	0.275
56	DPB1*01:01	−0.941
57	DPB1*02:01	1.112
58	DPB1*02:02	0.405
59	DPB1*03:01	0.493
60	DPB1*04:01	−1.238
61	DPB1*04:02	−0.438
62	DPB1*05:01	−0.408
63	DPB1*06:01	0.199
64	DPB1*09:01	1.043
65	DPB1*10:01	0.809
66	DPB1*11:01	0.007
67	DPB1*13:01	0.291
68	DPB1*14:01	0.223
69	DPB1*17:01	0.429
70	DPB1*19:01	−0.241
71	DQB1*02:01	−0.413
72	DQB1*02:02	0.197
73	DQB1*03:01	0.569
74	DQB1*03:02	−0.280
75	DQB1*03:03	−0.282
76	DQB1*04:02	−0.091
77	DQB1*05:01	−0.275
78	DQB1*05:02	0.560
79	DQB1*05:03	0.428
80	DQB1*06:01	0.267
81	DQB1*06:02	−0.696
82	DQB1*06:03	−0.767
83	DQB1*06:04	−0.662
84	DQB1*06:09	0.001
85	DRB1*01:01	−0.722
86	DRB1*01:02	0.540
87	DRB1*01:03	0.067
88	DRB1*03:01	0.494
89	DRB1*04:01	−1.363
90	DRB1*04:02	0.758
91	DRB1*04:03	0.524
92	DRB1*04:04	−0.278
93	DRB1*04:05	1.020
94	DRB1*04:07	0.248
95	DRB1*04:08	−0.341
96	DRB1*07:01	−0.007
97	DRB1*08:01	−0.104
98	DRB1*08:03	−0.057
99	DRB1*09:01	−0.131
100	DRB1*10:01	0.288
101	DRB1*11:01	0.847
102	DRB1*11:02	0.564
103	DRB1*11:03	0.820
104	DRB1*11:04	0.726
105	DRB1*12:01	−0.180
106	DRB1*13:01	−0.509
107	DRB1*13:02	−0.589
108	DRB1*13:03	0.821
109	DRB1*13:05	0.324
110	DRB1*14:01	0.361
111	DRB1*15:01	−1.353
112	DRB1*15:02	0.416
113	DRB1*16:01	0.544

**Table 3:** Pearson correlation coefficients between dementia risk scores and APR<1 values for the 9 HHV strains.

HHV Strain	Correlation coefficient	P value	N
1	0.063	0.567	85
2	−0.131	0.248	79
3	0.260**	0.013	91
4	−0.172	0.120	83
5	0.031	0.780	83
6A	0.025	0.840	67
6B	−0.007	0.951	89
7	0.287***	0.008	85
8	−0.038	0.719	90

## References

[1] Charonis S, James LM, Georgopoulos AP. In silico assessment of binding affinities of three dementia-protective Human Leukocyte Antigen (HLA) alleles to nine human herpes virus antigens. Curr Res Transl Med. 2020; 68(4): 211–6. 32624427 10.1016/j.retram.2020.06.002

[2] James LM, Georgopoulos AP. Immunogenetic epidemiology of dementia and Parkinson’s Disease in 14 Continental European countries: Shared human leukocyte antigen (HLA) profiles. J Immunological Sci. 2021; 5(2): 16–26. 10.29245/2578-3009/2021/2.1209 PMC12077081 40370814

[3] Kunkle BW, Grenier-Boley B, Sims R, Genetic meta-analysis of diagnosed Alzheimer’s disease identifies new risk loci and implicates Aβ, tau, immunity and lipid processing. Nat Genet. 2019; 51: 414–430. 30820047 10.1038/s41588-019-0358-2 PMC6463297

[4] Lambert JC, Ibrahim-Verbaas CA, Harold D, Meta-analysis of 74,046 individuals identifies 11 new susceptibility loci for Alzheimer’s disease. Nat Genet. 2013; 45(12): 1452–1458. 24162737 10.1038/ng.2802 PMC3896259

[5] Steele NZ, Carr JS, Bonham LW, Fine-mapping of the human leukocyte antigen locus as a risk factor for Alzheimer disease: a case–control study. PLOS Med. 2017; 14(3): e1002272. 28350795 10.1371/journal.pmed.1002272 PMC5369701

[6] Wang ZX, Wan Q, Xing A. HLA in Alzheimer’s Disease: Genetic association and possible pathogenic roles. Neuromol Med. 2020; 22: 464–473. 10.1007/s12017-020-08612-4 32894413

[7] Aliseychik MP, Andreeva TV, Rogaev EI. Immunogenetic factors of neurodegenerative diseases: The role of HLA class II. Biochemistry Moscow. 2018; 83: 1104–1116. 30472949 10.1134/S0006297918090122

[8] James LM, Georgopoulos AP. The human leukocyte antigen (HLA) DRB1*13:02 allele protects against dementia in continental Western Europe. J Neurol Neuromed. 2019; 4(5): 1–6. 10.29245/2572.942x/2019/5.1253 PMC12077165 40371383

[9] James LM, Georgopoulos AP. Tri-allelic human leukocyte antigen (HLA) protection against dementia. J Neurol Neuromed. 2020; 5(1): 12–17. 10.29245/2572.942x/2020/3.1275 PMC12077135 40371005

[10] James LM, Georgopoulos AP. Shared human leukocyte antigen (HLA) coverage in dementia and Parkinson’s disease. J Neurol Neuromed. 2020; 5(3): 45–54. 10.29245/2572.942x/2020/3.1275 PMC12077135 40371005

[11] Hov JR, Kosmoliaptsis V, Traherne JA, Electrostatic modifications of the HLA-DR P9 peptide-binding pocket and susceptibility to primary sclerosing cholangitis. Hepatology. 2011; 53: 1967–1976. 21413052 10.1002/hep.24299 PMC3128712

[12] Davenport MP, Quinn CL, Chicz RM, Naturally processed peptides from two disease-resistance-associated HLA-DR13 alleles show related sequence motifs and the effects of the dimorphism at position 86 of the HLA-DR beta chain. PNAS 1995; 92 (14): 6567–6571. 7604034 10.1073/pnas.92.14.6567 PMC41559

[13] Trowsdale J, Knight JC. Major histocompatibility complex genomics and human disease. Ann Rev Genom Hum Genet. 2013; 14: 301–323. 10.1146/annurev-genom-091212-153455 PMC4426292 23875801

[14] Simmonds MJ, Gough SC. The HLA region and autoimmune disease: Associations and mechanisms of action. Curr Genomics. 2007; 8(7): 453–65. 19412418 10.2174/138920207783591690 PMC2647156

[15] Maheshwari P, Eslick GD. Bacterial infection and Alzheimer’s disease: A meta-analysis. J Alzheimers Dis 2015; 43: 957–66. 25182736 10.3233/JAD-140621

[16] Mawanda F, Wallace R. Can infections cause Alzheimer’s disease? Epidemiol Rev 2013; 35: 161–80. 23349428 10.1093/epirev/mxs007 PMC4707877

[17] Itzhaki RF, Lathe R, Balin BJ, Microbes and Alzheimer’s disease. J Alzheimers Dis 2016; 51: 979–84. 26967229 10.3233/JAD-160152 PMC5457904

[18] Itzhaki RF. Corroboration of a major role for herpes simplex virus type 1 in alzheimer’s disease. Front Aging Neurosci. 2018; 10: e00324. 10.3389/fnagi.2018.00324 PMC6202583 30405395

[19] Chen VC, Wu SI, Huang KY, Herpes zoster and dementia: a nationwide population-based cohort study. J Clin Psychiat. 2018; 79: 16m11312. 10.4088/JCP.16m11312 29244265

[20] Eimer WA, Vijaya Kumar DK, Navalpur Shanmugam NK, Alzheimer’s disease associated beta-amyloid is rapidly seeded by Herpesviridae to protect against brain infection. Neuron. 2018; 99(1): 56–63. 30001512 10.1016/j.neuron.2018.06.030 PMC6075814

[21] Readhead B, Haure-Mirande JV, Funk CC, Multiscale analysis of independent Alzheimer’s cohorts finds disruption of molecular, genetic, and clinical networks by human herpesvirus. Neuron. 2018; 99(1): 64–82. 29937276 10.1016/j.neuron.2018.05.023 PMC6551233

[22] Westman G, Blomberg J, Yun Z, Decreased HHV-6 IgG in Alzheimer’s disease. Front Neurol. 2017; 8: 40. 28265256 10.3389/fneur.2017.00040 PMC5316842

[23] McQuillan G, Kruszon-Moran D, Flagg EW, Prevalence of herpes simplex virus type 1 and type 2 in persons aged 14–49: United States, 2015–2016. NCHS Data Brief, no 304. Hyattsville, MD: National Center for Health Statistics. 2018. 29442994

[24] Staras A, Dollard SC, Radford KW, Seroprevalence of cytomegalovirus infection in the United States, 1988– 1994. Clin Infect Dis 2006; 43: 1143–51. 17029132 10.1086/508173

[25] Wald A, Corey L, Persistence in the population: epidemiology, transmission. In: Arvin A, Campadelli-Fiume G, Mocarski E, editors. Human herpesviruses: biology, therapy, and immunoprophylaxis. Cambridge: Cambridge University Press; 2007 Chapter 36.

[26] The Uniprot Consortium 2019 (https://www.uniprot.org/).

[27] Janice S, Blum JS, Wearsch PA, Pathways of Antigen Processing. Annu Rev Immunol. 2013; 31: 443–73. 23298205 10.1146/annurev-immunol-032712-095910 PMC4026165

[28] Reynisson B, Alvarez B, Paul S, NetMHCpan-4.1 and NetMHCIIpan-4.0: improved predictions of MHC antigen presentation by concurrent motif deconvolution and integration of MS MHC eluted ligand data. Nucleic Acids Res. 2020; 48, W449–W454. 32406916 10.1093/nar/gkaa379 PMC7319546

[29] Breuer J, Pacou M, Gautier A, Herpes zoster as a risk factor for stroke and TIA: a retrospective cohort study in the UK. Neurology. 2014; 83(2): e27–33. 25002574 10.1212/WNL.0000000000000584

[30] Gilden D, Mahalingam R, Nagel MA, Review: The neurobiology of varicella zoster virus infection. Neuropathol Appl Neurobiol. 2011; 37(5): 441–63. 21342215 10.1111/j.1365-2990.2011.01167.x PMC3176736

[31] Gershon A, Breuer J, Cohen J, Varicella zoster virus infection. Nat Rev Dis Primers. 2015; 1: 1–18. 10.1038/nrdp.2015.16 PMC5381807 27188665

[32] Grahn A, Nilsson S, Nordlund A, Cognitive impairment 3 years after neurological Varicella-zoster virus infection: a long-term case control study. J Neurol. 2013; 260: 2761–2769. 23900759 10.1007/s00415-013-7057-1

[33] Chen VC, Wu SI, Huang KY, Herpes zoster and dementia: a nationwide population-based cohort study. J Clin Psychiat. 2017; 78(1): 16m11312. 10.4088/JCP.16m11312 29244265

[34] Tsai MC, Cheng WL, Sheu JJ, Increased risk of dementia following herpes zoster ophthalmicus. PloS One. 2017;12: e0188490. 29166672 10.1371/journal.pone.0188490 PMC5699837

[35] Bae S, Yun SC, Kim MC, Association of herpes zoster with dementia and effect of antiviral therapy on dementia: a population-based cohort study. Eur Arch Psychiatry Clin Neurosci. 2021; 271(5): 987–997. 32613564 10.1007/s00406-020-01157-4

[36] Lopatko Lindman K, Hemmingsson E-S, Weidung B, Herpesvirus infections, antiviral treatment, and the risk of dementia—a registry-based cohort study in Sweden. Alzheimer’s Dement. 2021; 7: e12119. 10.1002/trc2.12119 PMC7882534 33614892

[37] Helmuth IG, Poulsen A, Suppli CH, Varicella in Europe-A review of the epidemiology and experience with vaccination. Vaccine. 2015; 33(21): 2406–13. 25839105 10.1016/j.vaccine.2015.03.055

[38] James SF, Chahine EB, Sucher AJ, Shingrix: The new adjuvanted recombinant herpes zoster vaccine. Ann Pharmacother. 2018; 52(7): 673–680. 29457489 10.1177/1060028018758431

[39] Schnier C, Janbek J, Lathe R, Reduced dementia incidence following varicella zoster vaccination in Wales 2013–2020. medRxiv. 2021. doi: 10.1101/2021.07.22.21260981 PMC9006884 35434253

[40] Cermelli C, Fabio G, Montorsi M, Prevalence of antibodies to human herpesviruses 6 and 7 in early infancy and age at primary infection. New Microbiol 1996; 19: 1–8. 8673847

[41] Krueger GR, Koch B, Leyssens N, Comparison of seroprevalences of human herpesvirus-6 and −7 in healthy blood donors from nine countries. Vox Sang. 1998; 75: 193–7 9852406

[42] Dewhurst S, Skrincosky D, van Loon N. Human herpesvirus 7. Expert Rev Mol Med. 1997; 1(2): 1–10. 10.1017/S1462399497000112 14585127

[43] Wang FZ, Pellett PE. HHV-6A, 6B, and 7: immunobiology and host response. In: Arvin A, Campadelli-Fiume G, Mocarski E, Moore PS, Roizman B, Whitley R, Yamanishi K, editors. Human Herpesviruses: Biology, Therapy, and Immunoprophylaxis. Cambridge: Cambridge University Press; 2007. Chapter 48.

[44] Black JB, Pellett PE. Human herpesvirus 7. Rev Med Virol. 1999; 9(4): 245–62. 10578120 10.1002/(sici)1099-1654(199910/12)9:4<245::aid-rmv253>3.0.co;2-i

[45] Furukawa M, Yasukawa M, Yakushijin Y, Distinct effects of human herpesvirus 6 and human herpesvirus 7 on surface molecule expression and function of CD4+ T cells. J. Immunol 1994; 152: 5768–5775. 7911490

[46] Secchiero P, Gibellini D, Flamand L, Human herpesvirus 7 induces the down-regulation of CD4 antigen in lymphoid T cells without affecting p56lck levels. J. Immunol 1997; 159: 3412–3423. 9317140

[47] Glosson NL, Gonyo P, May NA, Insight into the mechanism of human herpesvirus 7 U21-mediated diversion of class I MHC molecules to lysosomes. J Biol Chem. 2010; 285(47): 37016–29. 20833720 10.1074/jbc.M110.125849 PMC2978630

[48] Tanaka-Taya K, Kondo T, Nakagawa N, Reactivation of human herpesvirus 6 by infection of human herpesvirus 7. J Med Virol. 2000; 60(3): 284–9. 10630960 10.1002/(sici)1096-9071(200003)60:3<284::aid-jmv6>3.0.co;2-8

[49] Yoshikawa T, Ihira M, Suzuki K, Invasion by human herpesvirus 6 and human herpesvirus 7 of the central nervous system in patients with neurological signs and symptoms. Arch Dis Child. 2000; 83(2): 170–1. 10906030 10.1136/adc.83.2.170 PMC1718426

[50] Tomsone V, Logina I, Millers A, Association of human herpesvirus 6 and human herpesvirus 7 with demyelinating diseases of the nervous system. J Neurovirol. 2001; 7: 564–569. 11704889 10.1080/135502801753248150

[51] Readhead B, Haure-Mirande JV, Funk CC, Multiscale Analysis of Independent Alzheimer’s Cohorts Finds Disruption of Molecular, Genetic, and Clinical Networks by Human Herpesvirus. Neuron. 2018; 99(1): 64–82. 29937276 10.1016/j.neuron.2018.05.023 PMC6551233

[52] Eimer WA, Kumar DK, Shanmugam NK, Alzheimer’s disease-associated β-amyloid is rapidly seeded by herpesviridae to protect against brain infection. Neuron. 2018; 99(1): 56–63. 30001512 10.1016/j.neuron.2018.06.030 PMC6075814

[53] Luo CS, Chi CC, Fang YA, Influenza vaccination reduces dementia in patients with chronic obstructive pulmonary disease: a nationwide cohort study. J Investig Med. 2020; 68: 838–845. 10.1136/jim-2019-001155 31941664

[54] Liu JC, Hsu YP, Kao PF, Influenza vaccination reduces dementia risk in chronic kidney disease patients: a population-based cohort study. Medicine. 2016; 95: e2868. 26945371 10.1097/MD.0000000000002868 PMC4782855

[55] Scherrer JF, Salas J, Wiemken TL, Lower risk for dementia following adult tetanus, diphtheria, and pertussis (Tdap) vaccination. J Gerontol Ser A. 2021; 76(8): 1436–1443. 10.1093/gerona/glab115 33856020

[56] Johnston C, Gottlieb SL, Wald A. Status of vaccine research and development of vaccines for herpes simplex virus. Vaccine. 2016; 34(26): 2948–52. 26973067 10.1016/j.vaccine.2015.12.076

[57] Buhler S, Sanchez-Mazas A. HLA DNA sequence variation among human populations: molecular signatures of demographic and selective events. PloS one. 2011; 6(2): e14643. 21408106 10.1371/journal.pone.0014643 PMC3051395

[58] Sanchez-Mazas A, Lemaître JF, Currat M. Distinct evolutionary strategies of human leucocyte antigen loci in pathogen-rich environments. Philos Trans R Soc Lond B Biol Sci. 2012; 367(1590): 830–9. 22312050 10.1098/rstb.2011.0312 PMC3267122

[59] Salamon H, Klitz W, Easteal S, Evolution of HLA class II molecules: Allelic and amino acid site variability across populations. Genetics. 1999; 152(1): 393–400. 10224269 10.1093/genetics/152.1.393 PMC1460587

